# Non-uniform Fourier transform based image classification in single-particle Cryo-EM

**DOI:** 10.1016/j.yjsbx.2025.100121

**Published:** 2025-02-03

**Authors:** ZiJian Bai, Jian Huang

**Affiliations:** University College Cork, Room 1-57, First Floor, Western Gateway Building, Western Road, Cork, T12 XF62, Ireland

**Keywords:** Non-uniform Fourier transform, 2D classification, Rotation-invariant feature extraction, Single-particle Cryo-EM

## Abstract

In the single-particle Cryo-EM projection image classification, it is a common practice to apply the Fourier transform to the images and extract rotation-invariant features in the frequency domain. However, this process involves interpolation, which can reduce the accuracy of the results. In contrast, the non-uniform Fourier transform provides more direct and accurate computation of rotation-invariant features without the need for interpolation in the computation process. Leveraging the capabilities of the non-uniform discrete Fourier transform (NUDFT), we have developed an algorithm for the rotation-invariant classification. To highlight its potential and applicability in the field of single-particle Cryo-EM, we conducted a direct comparison with the traditional Fourier transform and other methods, demonstrating the superior performance of the NUDFT.

## 1 Introduction

Single-particle electron microscopy (Cryo-EM) is a Nobel prize-winning technology to determine 3D structures of macromolecules at near-atomic resolution. With the rapid advancement of modern biological sciences, Cryo-EM has emerged as a powerful tool for elucidating the structures of bio-molecules (Nakane et al., 2020, Cheng, 2015). One of the major challenges in Cryo-EM is the unknown projection angles, combined with high noise levels due to the low electron dose required to avoid radiation damage. A key step in the 3D reconstruction process is to enhance the image signal-to-noise ratio (SNR) by averaging hundreds of thousands of projection images with the same projection angle (Bendory et al., 2020). This is achieved by classifying projection images with (nearly) identical projection angles into classes. While the projection images were obtained randomly which brings various types of translations, they may also contain images with poor imaging results like bad particles or overlapping. Thus 2D classification is a crucial step to analyse and distinguish images before aligning back the rotations and achieving class averaging (Zhou, 2008, van Heel and Schatz, 2017). Therefore, 2D image classification is a fundamental step in the entire 2D image processing pipeline, and its performance has a significant impact on the final reconstruction result (Serna, 2019, Sorzano et al., 2010).

A primary approach in addressing the 2D classification in single particle Cryo-EM is generating rotation-invariant features (Yin et al., 2019). Based on rotation invariant features generated from projection images, various methods have been developed to classify images with the same projection angle but different in-plane rotations into the same class (Zhou, 2008, Singer, 2018). The methods are different in the determination of rotation-invariant features. Cross-correlation and correntropy and more matrices have been used to measure the similarity between images (Schatz and Van Heel, 1990). Also, there are many mature methods in Cryo-EM software, the EMAN2 software (Tang et al., 2007) computes invariant features by computing the self-correlation function (SCF) of images (van Heel et al., 1992), followed by a polar transformation and then computing a 1D correlation on each ring to generate the final rotation invariant information. Xmipp software (Strelak et al., 2021, de la Rosa-Trevín et al., 2013) uses CL2D in the 2D class-averaging process, the algorithm is based on a hierarchical clustering approach, and it uses correntropy instead of conventional correlation to calculate the similarity between images. SPIDER (Frank et al., 1996) use reference-free alignment (Penczek et al., 1992) and then apply a rotational invariant K-means clustering (Penczek et al., 1996). Other methods including the use of spectral clustering (Wang et al., 2022) and maximum likelihood estimation (Stölken et al., 2011) also show effectiveness. Also, the Fourier transform has been used to extract rotation-invariant features, as the power spectrum obtained from the frequency domain in polar coordinates is rotation-invariant (Chen et al., 2021). However, the images are typically represented in a Cartesian coordinate system, a common approach is to use interpolation to convert frequency information in a Cartesian coordinate system to polar coordinates (for example, see Chen et al., 2021). The interpolation can introduce errors to the extracted rotation-invariant features or estimate in-plane rotation angles. In this study, we explore an alternative method that does not require interpolation.

To eliminate the need for interpolation and generate the rotation invariant information directly, we employ the NUDFT, a variant of the conventional Fourier transform. The NUDFT enables the generation of the Fourier transform at a desired and specified frequency point (Fessler and Sutton, 2003). This capability gives us the flexibility to calculate power spectrum frequency points in the polar format, thereby converting the rotation information from the Cartesian coordinate system to the polar coordinate system, aligning with our initial requirements in 2D classification. In this paper, we present a method based on the NUDFT and thoroughly investigate its performance by comparing it with the traditional Fourier transform approach. We expect that this analysis will provide a fresh perspective on the 2D classification problem in Cryo-EM.

## 2 Method

### 2.1 Non-uniform discrete Fourier transform (NUDFT)

The Fourier transform is a mathematical tool that transforms a signal from the time domain to the frequency domain and decomposes the signal into a sum of sine and cosine functions. It typically assumes that the signal is obtained under uniform sampling, i.e. with a fixed sampling interval. In practical applications, however, non-uniform sampling may occur, and this is where the non-uniform Fourier transform (NUFT) comes into play.

The NUFT is a variant of the Fourier transform that can handle non-uniformly sampled data and generate non-uniformly sampled frequency information (Fessler, 2001). Unlike the traditional Fourier transform, NUFT does not require equidistant sampling in the time domain or frequency domain (Dutt and Rokhlin, 1993). Instead, it can process data with irregular sampling point distributions and is suitable for a variety of applications.

NUFT comes in three types: Type 1 NUFT transforms uniformly sampled signals to the non-uniformly sampled frequency domain, while Type 2 NUFT transforms non-uniformly sampled signals to the uniform frequency domain, and Type 3 NUFT transforms non-uniformly sampled signals to the non-uniformly sampled frequency domain.

The 2D Type 1 NUDFT is a variant of the Type 1 NUFT that is used to convert a two-dimensional signal or image from uniformly sampled data to a non-uniformly sampled frequency domain.

The 2D Type 1 NUDFT is defined as:

(1) $$F\left(u,v\right)=\overset{N-1}{\underset{x=0}{\sum }}\overset{M-1}{\underset{y=0}{\sum }}f\left(x,y\right)\cdot e^{-i2\pi \left(\frac{ux}{N}+\frac{vy}{M}\right)}$$

Where F(u,v) represents the signal in the frequency domain (two-dimensional Fourier domain), where (u,v) is the frequency domain coordinate. f(x,y) represents the signal in the time domain (two-dimensional space domain), while (x,y) is the coordinate of the uniform sampling point. N represents the number of sampling points in the x direction. M represents the number of sampling points in the y direction.

In summary, the key difference between the 2D discrete Fourier transform and 2D Type 1 NUDFT is that the latter is capable of generating frequency information with non-uniform distribution while the former requires equidistant distribution.

### 2.2 Rotation invariant information

Rotation-invariant information of the image can be obtained from the frequency information generated from the Fourier transform. When applied to an image, the Fourier transform converts the image from the spatial domain to the frequency domain, providing us with information about the intensity and phase of different frequency components of the image. The power spectrum of Fourier transform is a term used to describe the distribution of signal power as a function of frequency, it represents the amount of energy present in each frequency component of a signal.

The power spectrum is particularly useful in extracting rotation-invariant information in image analysis (Lucchese and Cortelazzo, 2000). As demonstrated in the following sections, the power spectrum of the Fourier transform in polar coordinates does not contain information about the orientation or rotation of the image. Thus, we can use the power spectrum to extract rotation-invariant features (information), which can be used for further image analysis and pattern recognition tasks.

Considering two images f and g with g being the in-plane $\theta_{0}$ rotated version of f. Under Cartesian coordinates we can have:

(2) $$G\left(u,v\right)=F\left(u\cos\left(\theta_{0}\right)-v\sin\left(\theta_{0}\right),u\sin\left(\theta_{0}\right)+v\cos\left(\theta_{0}\right)\right)$$

This implies the Fourier transform of the in-plane rotated version G(u,v) and the Fourier transform of the original F(u,v) have the same rotation relationship between f(x,y) and g(x,y) with in-plane rotation angle $\theta_{0}$.

According to the NUFT property, we can have u,v set in the format converted from ω and φ (which is in polar format), and the original Cartesian coordinate system in the original image f now has corresponding r, θ in the polar coordinate system. Then we have:

(3) $$\left\{\begin{array}{c} x=r\cdot \cos\left(\theta \right)\;\\ y=r\cdot \sin\left(\theta \right)\; \end{array}\right)\left\{\begin{array}{c} u=\omega \cdot \cos\left(\varphi \right)\;\\ v=\omega \cdot \sin\left(\varphi \right)\; \end{array}\right)$$

Now, the NUDFT formula can be written as:

(4) $$F\left(\omega ,\varphi \right)=\overset{N-1}{\underset{r=0}{\sum }}\overset{2\pi }{\underset{\theta =0}{\sum }}f\left(r,\theta \right)\cdot e^{-i\cdot 2\pi \cdot \left(\omega \cdot r\cdot \cos\left(\varphi \right)\cdot \cos\left(\theta \right)+\omega \cdot r\cdot \sin\left(\varphi \right)\cdot \sin\left(\theta \right)\right)}$$

(5) $$F\left(\omega ,\varphi \right)=\overset{N-1}{\underset{r=0}{\sum }}\overset{2\pi }{\underset{\theta =0}{\sum }}f\left(r,\theta \right)\cdot e^{-i\cdot 2\pi \cdot \omega \cdot r\cdot \cos\left(\theta -\varphi \right)}$$

And $x^{\prime }$, $y^{\prime }$ in image g now convert into $r^{\prime }$, $\theta^{\prime }$, also, $u^{\prime }$, $v^{\prime }$ have its corresponding $\omega^{\prime }$, $\varphi^{\prime }$ in polar coordinate system as well. Thus we can have:

(6) $$\left\{\begin{array}{c} \theta^{\prime }=\theta +\theta_{0}\;\\ \theta =\theta^{\prime }-\theta_{0}\; \end{array}\right)$$

And the NUDFT of g can be written as:

(7) $$G\left(\omega ,\varphi^{\prime }\right)=F\left\{g\left(r^{\prime },\theta^{\prime }\right)\right\}=F\left\{f\left(r,\theta +\theta_{0}\right)\right\}$$

(8) $$G\left(\omega ,\varphi^{\prime }\right)=\overset{N-1}{\underset{r=0}{\sum }}\overset{2\pi }{\underset{\theta =0}{\sum }}f\left(r,\theta +\theta_{0}\right)\cdot e^{-i\cdot 2\pi \cdot \omega \cdot r\cdot \cos\left(\theta -\varphi \right)}$$

(9) $$G\left(\omega ,\varphi^{\prime }\right)=\overset{N-1}{\underset{r=0}{\sum }}\overset{2\pi }{\underset{\theta^{\prime }=0}{\sum }}f\left(r,\theta^{\prime }\right)\cdot e^{-i\cdot 2\pi \cdot \omega \cdot r\cdot \cos\left(\theta^{\prime }-\theta_{0}-\varphi \right)}$$

(10) $$G\left(\omega ,\varphi^{\prime }\right)=\overset{N-1}{\underset{r=0}{\sum }}\overset{2\pi }{\underset{\theta^{\prime }=0}{\sum }}f\left(r,\theta^{\prime }\right)e^{-i\cdot 2\pi \cdot \omega \cdot r\cdot \cos\left[\theta^{\prime }-\left(\varphi +\theta_{0}\right)\right]}$$

Then we can have:

(11) $$G\left(\omega ,\varphi^{\prime }\right)=F\left(\omega ,\varphi +\theta_{0}\right)$$

Thus, by setting the frequency points in polar system format during NUDFT, the rotation relationship between images f and g now converts into a translation relationship among angular frequency. The corresponding amplitudes (or power spectrums) obtained by applying a 1D Fourier along angular frequency will be the same.

Now consider applying a 1D Fourier transform on all angular frequency components at each radial frequency. Considering F(s) and G(s) as the Fourier transform of Amplitude obtained from prior NUDFT, then we can have:

(12) $$F_{j}\left(s\right)=F_{1D}\left\{\left|F\left(\omega_{j},\varphi_{t}\right)\right|\right\}=\overset{N-1}{\underset{t=0}{\sum }}\left|F\left(\omega_{j},\varphi_{t}\right)\right|\cdot e^{-i\cdot 2\pi \cdot s\cdot \varphi_{t}}$$

(13) $$G_{j}\left(s\right)=F_{1D}\left\{\left|F\left(\omega_{j},\varphi_{t}^{\prime }\right)\right|\right\}$$

Let $\varphi_{t}^{\prime }=\varphi_{t}+\theta_{o}$, then we have:

(14) $$G_{j}\left(s\right)=\overset{N-1}{\underset{t=0}{\sum }}\left|F\left(\omega_{j},\varphi_{t}^{\prime }\right)\right|\cdot e^{-i\cdot 2\pi \cdot s\cdot \left(\varphi_{t}^{\prime }-\theta_{0}\right)}=e^{i\cdot 2\pi \cdot s\cdot \theta_{0}}\cdot \overset{N-1}{\underset{t=0}{\sum }}\left|F\left(\omega_{j},\varphi_{t}^{\prime }\right)\right|\cdot e^{-i\cdot 2\pi \cdot s\cdot \varphi_{t}^{\prime }}$$

Since $\left|e^{i\cdot 2\pi \cdot s\cdot \theta_{0}}\right|=1$, then we can have:

(15) $$\left|G_{j}\left(s\right)\right|=\left|F_{j}\left(s\right)\right|$$

Thus we can recognize this amplitude (or further compute the power spectrum) as the rotation-invariant information of the images.

In summary, amplitude (and the power spectrum) is a fundamental concept in the Fourier transform that is useful in addressing the problem of rotation angle classification in image analysis (Bendory et al., 2020). By analysing the amplitude of an image and combining the unique property of the NUDFT, we can obtain rotation information of the given images and thus serving as a satisfied computational basis and facilitating further analysis.

### 2.3 Principal Component Analysis(PCA)

Principal Component Analysis (PCA) is a highly effective technique for dimension reduction (Labrín and Urdinez, 2020). In our particular case, we have applied the NUDFT, which has provided us with frequency information representing rotation-invariant features in the frequency domain. To establish a rotation-invariant distance metric between the projection images derived from these large-sized data matrices, dimension reduction becomes crucial to simplify the computational complexity.

The rationale for employing PCA as the preferred dimension reduction technique in this context stems from its suitability in handling computational frequency information. The NUDFT yields polar-distributed frequency information rather than the original image intensities. As a result, the data matrix now represents the original image in the frequency domain. Known that frequency information often exhibits significant contributions in the lower frequency range while minor contributions in other frequencies, and rotation-invariant decisions are partly based on these discrepancies, we aim to preserve this relationship as much as possible during the dimension reduction process. Moreover, the computation process of PCA possesses the ability to effectively reduce the noise present in the information as well as eliminate redundant information pertaining to certain aspects of the frequency information (Shlens, 2014). This characteristic contributes to the enhancement of performance in subsequent cluster processes.

### 2.4 K-means clustering

The rotation invariant features calculated by the NUDFT serve the purpose of solving a 2D classification problem. Our objective is to perform this classification task solely based on the features derived from different projection images. Hence, an unsupervised clustering method is needed to classify all the rotation-invariant features.

In this context, K-means Clustering emerges as a suitable approach due to its simplicity and efficiency. K-means clustering operates by iteratively assigning data points to the cluster with the nearest mean, and then updating the cluster means based on the newly assigned points (Hartigan and Wong, 1979). In our case, we use Euclidean distance between each generated principal component (PC) to describe the similarity matrix included in the computational process, and these cluster-means represent the centroids of the rotation-invariant features, which are indicative of the representative characteristics of each cluster. By employing K-means clustering, we can automatically group the rotation-invariant features into distinct clusters without any prior knowledge of the class labels. This allows us to perform the classification task solely based on the similarities and dissimilarities between the features, enabling an efficient and effective solution to our 2D classification problem.

Thus, K-means clustering is well-suited for this classification task as it leverages the mean value calculation to distinguish different frequency information, aligning with the features obtained from the NUDFT.

### 2.5 Algorithm flow

The Fourier transform is a powerful tool for analysing the intensity values of an image in the frequency domain. In contrast, the NUDFT provides greater flexibility in choosing the distribution of frequency points of interest. By representing these points in polar coordinates, we can examine the power spectrum under the frequency domain and generate rotation-invariant information that is useful for distinguishing images just using basic techniques. We conducted a comparison among NUDFT and 2DFFT and CL2D used in the Xmipp software for the classification problem. Our goal was to evaluate their performance by developing algorithms for these methods and conducting the experiments under identical conditions while the only difference is the computation basis. This approach allowed us to observe and quantify the ability and advantages of NUDFT directly. We anticipate discovering intriguing results and gaining insights specifically from the NUDFT analysis.

First, we developed an algorithm for NUDFT. Prior to applying the Fourier transform, it is important to select a region of interest within the projection image. In general, the structure of molecules is concentrated in the centre of the image, while the four corners contain only noise information. Thus, we choose a circular region with the diameter set as the width of the square projection image.

After obtaining a circular mask of the original projection image, we apply the NUDFT. By considering the centre of the circle as the origin of the polar coordinate system, we can set all frequency coordinates generated in NUDFT in a polar format, which facilitates further computation. During the Fourier transform, we represent the original information under the frequency domain.

After applying the NUDFT to the projection images, we obtain the power spectrum for subsequent computation. The rotation relationship between images now converts into the translation relationship among angular directions. Since the frequency points are in polar format, we apply the Fast Fourier transform to the power spectrum along the angle direction to offset the rotation factor. Consequently, the power spectrum derived from this FFT yields rotation-invariant information, serving as a novel representation obtained from the original projection images.

**Figure:**
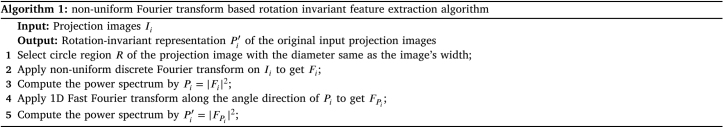

In order to facilitate a comprehensive comparison between the NUDFT and the conventional Fourier transform in addressing the given problem, we devised a straightforward algorithm for the 2DFFT. This algorithm incorporates interpolation during the conversion of frequency information to the Polar format, enabling the analysis of rotation-invariant information. These distinctions provide a direct basis for contrasting the NUDFT with the 2DFFT, while the prior process eliminates the need for interpolation in the Polar transform.

To extract rotation-invariant information from the frequency information obtained via the conventional 2D Fourier transform, a preliminary step involves applying an FFT shift, which rearranges the Fourier transform by shifting the zero-frequency component to the centre of the matrix. Consequently, the modified frequency information retains the same rotation as the original projection images. Subsequently, we derive the power spectrum from the frequency information for further analysis. By employing a Polar transform, we convert the power spectrum into Polar coordinates. Later, an additional 1D FFT is performed along the angle direction for each radius, thereby offsetting the in-plane rotation embedded in the frequency information. The resulting power spectrum obtained from the 1D FFT yields the rotation-invariant information from the original projection images. Also, we conduct zero-padding for 2DFFT, as zero-padding in the image domain can extend the dimension of image frequency information and then facilitate the interpolation process. We include zero-padding based 2DFFT as a benchmark to evaluate the performance of the proposed NUDFT. As our primary focus lies on contrasting the performance of Fourier transform methods.

**Figure:**
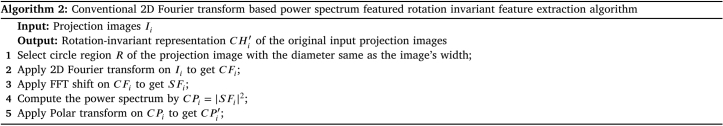

Following the acquisition of rotation-invariant information from the projection images through NUDFT processes, it becomes necessary to perform additional modifications. The data matrix is characterized by a large size, resulting in a substantial computational burden. Furthermore, the presence of noise and redundancy within certain sections of the data can potentially impact the clustering outcome. Consequently, a dimension reduction process is imperative. While the rotation invariant features obtained have been computed from a NUDFT process following a 1D Fourier transform among angular direction’s frequency components, the amplitude generated from the last Fourier transform now represents the rotation invariant information under each radial component, thus the application of PCA can be performed by regarding each radial component’s rotation invariant information as a factor.

Subsequently, we apply the K-means clustering method to classify the rotation-invariant information after dimension reduction and generate distinct classes from the entirety of the projection images. This approach is more conducive to the analysis of the results derived from both Fourier transform processes.

**Fig. 1:**
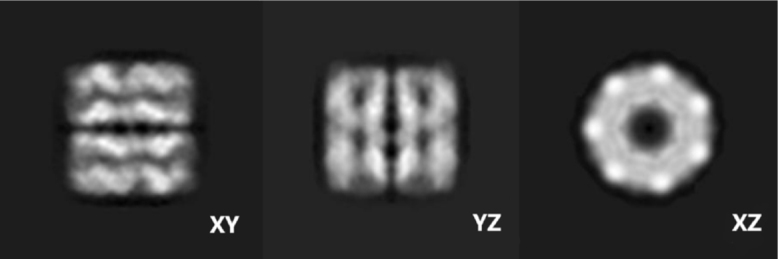
EMDB 1080.

## 3 Experimental results

### 3.1 Experimental results on a simulated Cryo-EM dataset

**Synthetic data generation**

**Fig. 2:**
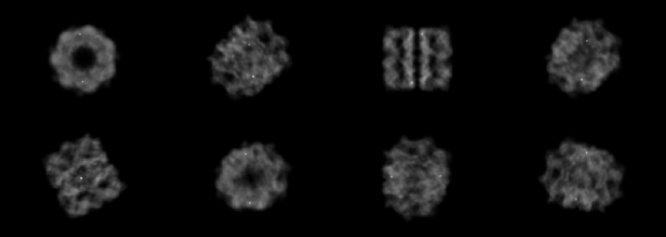
Different classes of projection images are specified by different projection angles.

We generated projection images from EMDB 1080 (Ludtke et al., 2001), a public real-world GroEL dataset, with size 256 × 256 (slice projection image shown in Fig. 1). 8 distinct class centres were thoughtfully selected to represent different projection angles, and within each class, 32 images were constructed to simulate the in-plane rotation by π/16 (different class centres are shown in Fig. 2). As a result, a comprehensive set of 256 projection images was successfully constructed, effectively simulating the clustering problem basis before applying image computational processes.

We simulate the noise environment by adding noise to the clean image generated prior. Since we already know the 3D structure, we can calculate the variance of the structure to create and add noise according to different SNR requirements. Our SNR is defined based on variance:

(16) $$SNR=\frac{Var\left(Signal\right)}{Var\left(Noise\right)}$$

In addition, unlike traditional noise that only uses Gaussian noise, different types of noise may appear during the imaging process, like auto-correlated noise (Reimer, 2013, Fultz and Howe, 2012). Thus, we use auto-correlated noise to simulate different types of distributed noise that may appear in the imaging process, and use this to test how the NUDFT algorithm handles this type of noise. In our case, the noise sequence is constructed using an order 2 auto-regressive (AR (2)) model:

(17) $$X_{t}=\phi_{1}X_{t-1}+\phi_{2}X_{t-2}+{ɛ}_{t}$$

**Fig. 3:**
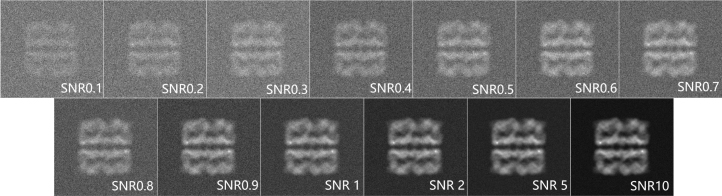
Synthetic projection images were constructed under different signal-to-noise ratios.

This means the third value of the sequence is determined by the first two values, while our case is in 2D scenarios, the centre of the image is considered as the origin and the AR sequence is distributed along radius. Thus, we set the noise variance according to the required SNR and then add them to the clean projection image to finally generate noisy images with different SNR levels. We constructed noise under different SNRs ranging from 0.1 to 10 (shown in Fig. 3).

**PCA and K-means classification result examination**

**Fig. 4:**
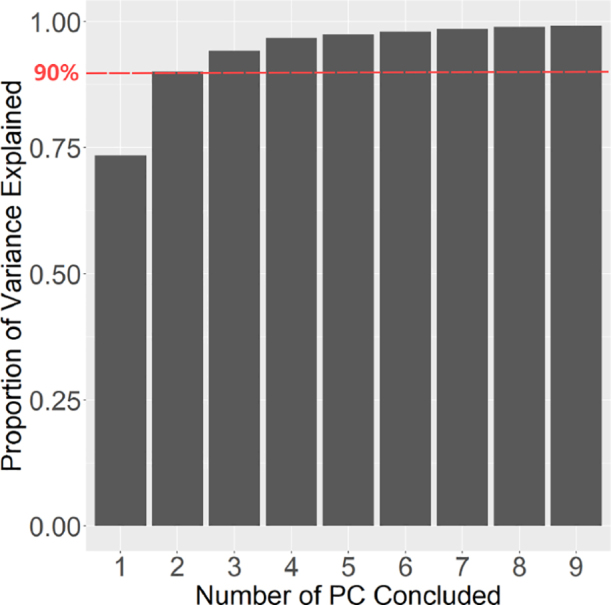
The cumulative variance explained ratio for different numbers of PC concluded. The variance explained ratio indicates the proportion of the total variance in the original dataset that is captured by each principal component in PCA. The accumulated variance explained ratio measures the ability of the first N principal components to retain the original information during dimensionality reduction.

The computed rotation-invariant representations derived from the NUDFT method still maintain a relatively high dimensionality, leading to substantial computational overhead and potential confusion during subsequent classification procedures. To address these challenges, we employed PCA to perform dimensionality reduction on the data. In our case, the frequency information generated from the NUDFT algorithm has dimensions of 80 × 256, corresponding to 80 radial components and 256 angular components in the NUDFT (we set these numbers based on our different attempts), then the principle components generated from the PCA has dimensions of 80 × 80. This reduction aimed to streamline the dataset’s dimensions, resulting in improved computational efficiency. Variance explained ratios of each principle component in our case are shown in Fig. 4. By extracting the first two principal components, we effectively preserved vital and informative attributes from the original dataset while achieving a reduced-dimensional representation.

Subsequent to the application of dimension reduction through PCA, an unsupervised classification approach was implemented, utilizing the k-means clustering algorithm. The primary objective of employing k-means clustering was to partition the dataset’s data points into well-defined clusters, guided by the resemblance patterns extracted from the reduced-dimensional representation acquired through PCA. In our case, we concluded the first two components (which have dimensions of 2 × 80) of each image in the K-means process, and we regarded these PCs as a long vector, thus each image’s rotation invariant features concluded in the K-means clustering has dimensions of 1 × 160, finally, we use Euclidean distance to compute the difference between each images’ rotation invariant features(now we have transformed the original 80 × 256 frequency information into 1 × 160 length vector). The distance metrics we used in K-means clustering is the Euclidean distance between each images’ features:

(18) $$\mathrm{EucDist}\left(x,y\right)=\sqrt{{\left(x-y\right)}^{⊤}\left(x-y\right)}=\sqrt{\overset{D}{\underset{i=1}{\sum }}{\left(x_{i}-y_{i}\right)}^{2}}$$

While x,y is each image’s rotation invariant features, in our case, it is a vector.

**Fig. 5:**
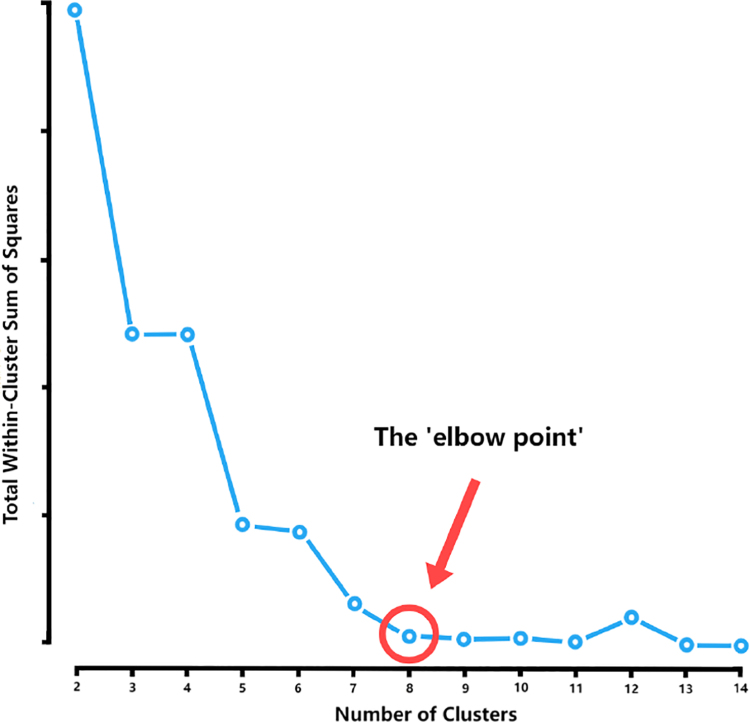
Within-Cluster sum of squares under different numbers of clusters.

In this unsupervised clustering task, we were also mindful of the potential influence of noise factors, which introduced a level of uncertainty regarding the appropriate number of clusters at the commencement of the computational process. Consequently, we resorted to the elbow point method as a principled technique to ascertain the optimal number of clusters for the k-means algorithm. This method entails an assessment of the within-cluster sum of squares (shown in Fig. 5) across a range of candidate cluster numbers. The pivotal ‘elbow point’ signifies a juncture where the pace of reduction in the sum of squares experiences a notable deceleration. This inflexion point is indicative of a potential optimal cluster count that attains a balance between model complexity and clustering fidelity. As delineated in Fig. 5, the number of clusters suggested by the ‘elbow point’ harmonizes notably well with the ground truth number of clusters. Consequently, we embarked on a subsequent phase of rigorous scrutiny, assessing the classification accuracy vis-à-vis the identified clusters. Furthermore, we conducted a comparison of classification performance by leveraging the rotation invariant representations computed via these distinct methods.

This multifaceted analytical exploration encompasses a comprehensive examination of the underlying structure, noise resistance, and information retention achieved through the synergy of dimension reduction and unsupervised clustering. The comparison of the ‘elbow point’ indication and the true cluster count substantiates the efficacy of the elbow method within the context of unsupervised classification scenarios, thereby enhancing our confidence in the subsequent classification assessments conducted.

**Classification result comparison**

**Table 1:** Classification accuracy of each method in synthetic data set.

SNR	Xmipp	NUDFT	2DFFT	2DFFT-ZP
10	0.9531	1.0000	0.9531	1.0000
5	0.9062	1.0000	0.9531	1.0000
2	0.8906	0.9688	0.9531	0.9688
1	0.8750	0.9688	0.9375	0.9688
0.9	0.8594	0.9688	0.9375	0.9531
0.8	0.8594	0.9531	0.9062	0.9375
0.7	0.8594	0.9375	0.8906	0.9219
0.6	0.8281	0.9375	0.8750	0.9062
0.5	0.8125	0.9219	0.8438	0.8906
0.4	0.7812	0.9062	0.7969	0.8750
0.3	0.7031	0.8906	0.7812	0.8438
0.2	0.6719	0.8906	0.7656	0.8125
0.1	0.6250	0.8750	0.7344	0.7812

In order to assess and compare the efficacy and robustness of noise of the developed algorithms on NUDFT processes, we conducted a rigorous evaluation of a real-world particle with different signal-to-noise ratios. This evaluation involved subjecting the algorithms to constructed datasets featuring different levels of noise, simulating SNRs ranging from 10 to 0.1 (shown in Fig. 3).

As we construct projection images from a pre-known 3D structure, we know each image’s correct class (or called category) initially, thus when we compute the clustering accuracy we can use a more precise and more realistic way to define the correctly classified images. In simple terms, the accuracy can be written as:

(19) $$\text{Accuracy}=\frac{\text{Number of Correctly Classified Samples}}{\text{Total Number of Samples}}$$

**Fig. 6:**
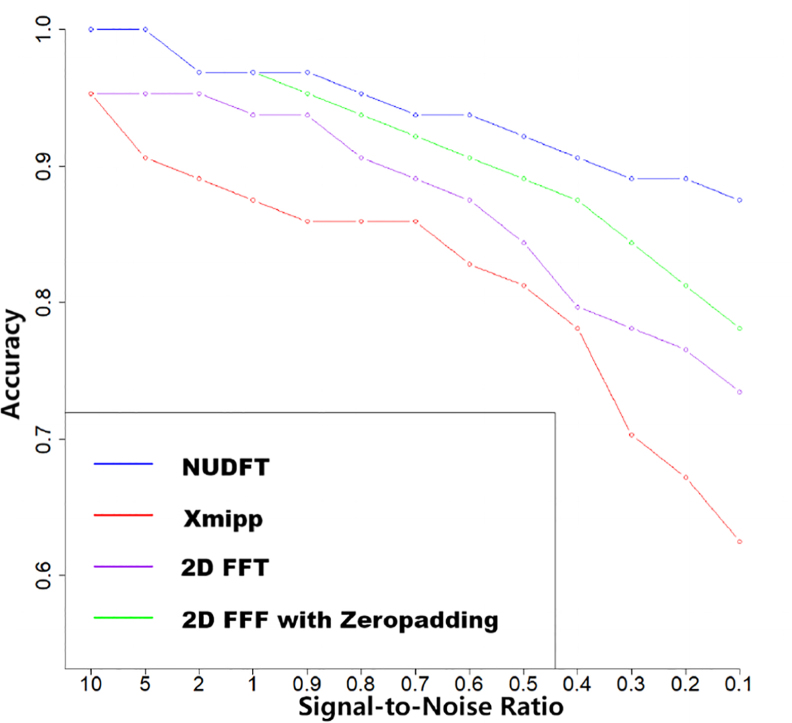
Classification accuracy of different methods. The blue line shows the accuracy of the NUDFT method, the red line shows Xmipp’s method, the purple line shows the 2DFFT and the green line shows the 2DFFT with zero-padding enhancing its interpolation process. (For interpretation of the references to colour in this figure legend, the reader is referred to the web version of this article.)

By conducting such a comprehensive evaluation(results shown in Fig. 6 and Table 1), we sought to draw conclusive insights regarding the comparative performance of the NUDFT processes. Based on the experimental results presented in Table 1, it is evident that the NUDFT algorithm outperforms the 2DFFT and Xmipp algorithms in terms of overall signal-to-noise ratios. Although the performance of each method is acceptable at high SNR conditions, the accuracy of Xmipp has a great influence as the SNR becomes lower. And the result proved that zero-padding can improve the accuracy of the Fourier transform as it benefits the interpolation process, but NUDFT still shows better capability in the overall performance while it does not conclude the zero-padding process. The NUDFT algorithm demonstrates enhanced robustness against noise and exhibits greater stability across varying levels of SNRs. Notably, in cases of higher SNR, the NUDFT algorithm exhibits a remarkable superiority in classification accuracy.

### 3.2 Experimental results on a real Cryo-EM dataset

**dataset description**

**Fig. 7:**
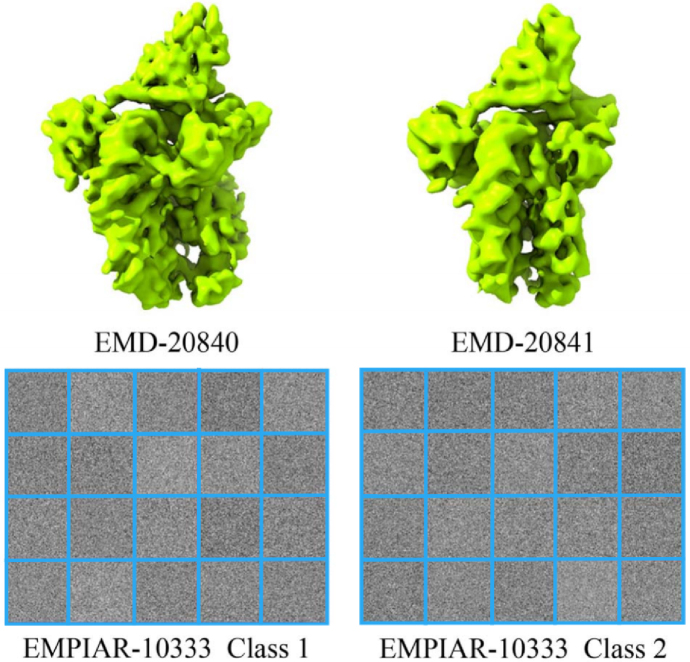
EMPIAR-10333. EMPIAR-10333 contains EMD-20840 and EMD-20841. The EMPIAR-10333 dataset has been used to reconstruct the published Cryo-EM 3D density maps of FACT sub-nucleosome complex 1 (EMD-20840) and FACT sub-nucleosome complex 2 (EMD-20841) from these two classes of projection images.

We perform the comparison on a real heterogeneous Cryo-EM dataset containing projection images randomly chosen from the extracted particle images of the human facilitates chromatin transcription (FACT) in complex with partially assembled sub-nucleosomes (EMPIAR-10333) (Liu et al., 2020). The EMPIAR-10333 dataset has been used to reconstruct the published Cryo-EM 3D density maps of FACT sub-nucleosome complex 1 (EMD-20840) and FACT sub-nucleosome complex 2 (EMD-20841) from these two classes of projection images (shown in Fig. 7), they have very similar structure which is suitable in performing classification. As the full EMPIAR-10333 dataset has been divided into two classes, we randomly chose 1000 projection images from two classes, thus the real dataset contains 2000 projection images. The size of projection images is 200 × 200.

**PCA and K-means result examination**

**Fig. 8:**
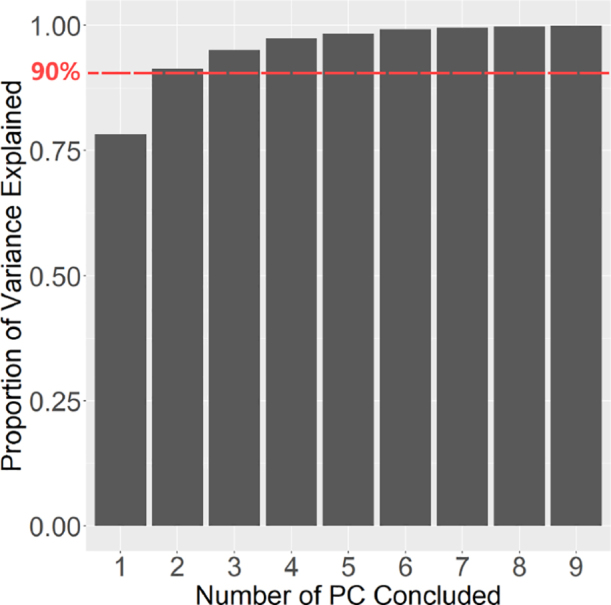
The cumulative variance explained ratio for different numbers of PC concluded.

**Fig. 9:**
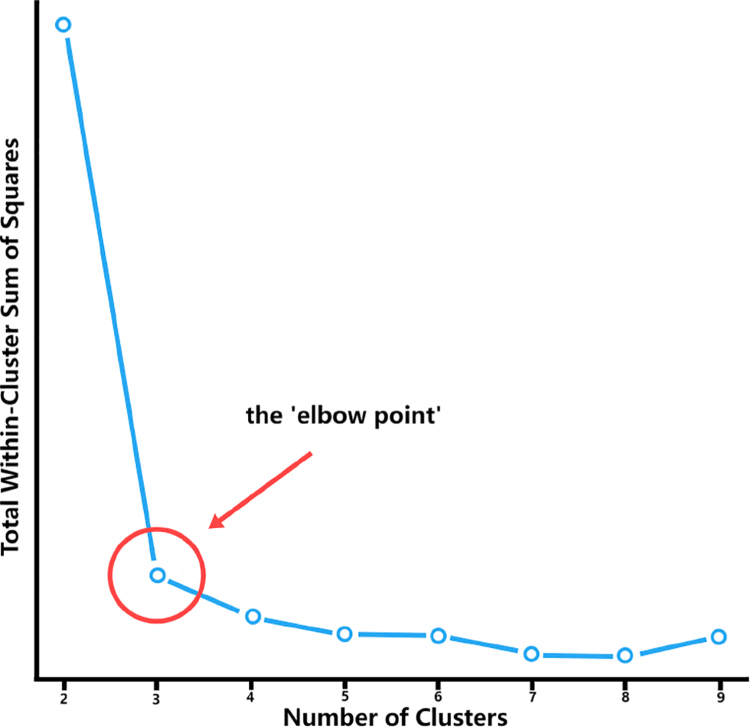
Within-Cluster sum of squares under different numbers of clusters.

As shown in Fig. 8, the first two PC can preserve much of the variation of the frequency information generated from the NUDFT process, this result is consistent with the synthetic data’s result and our motivation. Also, the ‘elbow point’ in K-means clustering (shown in Fig. 9) also shows satisfied results as the number of clusters generated from this unsupervised clustering is very close to the ground truth of the data, proving the effectiveness of the application of the K-means clustering.

**Classification result comparison**

**Fig. 10:**
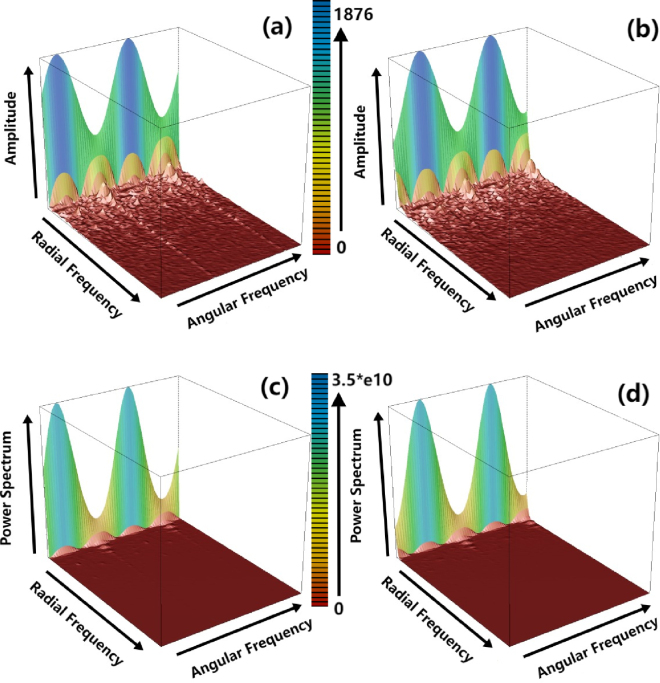
3D histogram of the amplitude and power spectrum generated from our proposed algorithm. (a) and (b) is the amplitude and power spectrum of one image while (c) and (d) are generated from a different class, (a) (b) and (c) (d) both show the clear difference between the two images, the peak of the amplitude (and spectrum) shows the rotational transformation is converted to the translational transformation, the lag along angular direction can be used to determine the rotation angle directly, and the rotation invariant information generated by amplitude and power spectrum both valid.

Since the projection images in the real dataset are randomly chosen from EMPIAR-10333 Class 1 and EMPIAR-10333 Class 2, thus we know the ground truth of the classification result. After applying our proposed NUDFT algorithm (NUDFT result shown in Fig. 10), then we compute the accuracy for our proposed NUDFT algorithm, CL2D, 2DFFT and 2DFFT with zero-padding. As shown in the result (Table 2), the algorithm using Fourier transform can have better results than CL2D, and our proposed NUDFT algorithm shows enhanced performance compared to 2DFFT.

**Operation time summary**

**Table 2:** Classification accuracy of each method in real-world data sets.

Xmipp	NUDFT	2DFFT	2DFFT-ZP
0.5110	0.6425	0.6075	0.6235

All experiments were performed on an AMD Ryzen 9 5900HX processor with 32 GB memory. The CL2D method takes 104267 s, and our proposed NUDFT based method takes 90275 s, the 2D FFT method takes 11246 s, and the 2D FFT with zero-padding method takes 14137 s. Though NUDFT takes longer runtime compared with 2D FFT, it performs overall better accuracy according to our experimental result.

## 4 Summary and discussion

As discussed in the previous section, the NUDFT has demonstrated superior performance in the overall comparison, displaying enhanced noise resistance and performance under different SNR and real-world datasets, particularly in low signal-to-noise ratio environments. These enhanced capabilities are attributed to its inherent property of deriving frequency information at any specified interest frequency point, allowing it to generate frequency information and extract rotation information directly, bypassing the interpolation step which avoids additional errors, which result in its robustness to noise and overall superior performance. Furthermore, it provides the flexibility to generate more frequency information for any given frequency domain and its unique property also grants it superior performance in handling auto-correlated noise.

Although our comparison is based on a limited number of data sets, the results clearly highlight the differences in the NUDFT process. We aim to emphasize the significance of NUDFT and its potential in addressing Cryo-EM rotation problems. By renewing the interest in NUDFT, we can further explore its capabilities and contributions to Cryo-EM research.

## CRediT authorship contribution statement

**ZiJian Bai:** Writing – original draft, Visualization, Validation, Methodology, Investigation, Data curation. **Jian Huang:** Writing – review & editing, Supervision, Methodology, Investigation, Formal analysis, Data curation, Conceptualization.

## Declaration of competing interest

The authors declare that they have no known competing financial interests or personal relationships that could have appeared to influence the work reported in this paper.
